# Effects of a 217‐km mountain ultramarathon on the gut microbiota of an obese runner: A case report

**DOI:** 10.14814/phy2.70017

**Published:** 2024-08-22

**Authors:** Giulio Kai Saragiotto, Luiz Felipe Valter de Oliveira, Nayara Kastem Scharlack, Milena Merizzi de Oliveira, Fernanda Campos Freire, Fernando Moreira Simabuco, Katia Sivieri, Adilson Sartoratto, Taisa Belli, Adriane Elisabete Costa Antunes

**Affiliations:** ^1^ Faculdade de Ciências Aplicadas Universidade Estadual de Campinas (FCA/UNICAMP) Limeira São Paulo Brazil; ^2^ Department of Biotechnologies BiomeHub Florianópolis Santa Catarina Brazil; ^3^ School of Pharmaceutical Sciences, State University of São Paulo “Júlio de Mesquita Filho” (UNESP) Araraquara São Paulo Brazil; ^4^ Departamento de Bioquímica Universidade Federal de São Paulo (UNIFESP) São Paulo São Paulo Brazil; ^5^ Centro Pluridisciplinar de Pesquisas Químicas Biológicas e Agrícolas, Universidade Estadual de Campinas (CPQBA/UNICAMP) Paulínia São Paulo Brazil; ^6^ Departamento de Ciências do Esporte Faculdade de Educação Física, Universidade Estadual de Campinas (FEF/UNICAMP) Campinas São Paulo Brazil

**Keywords:** gut microbiota, obese runner, pathobionts bacteria, ultramarathon

## Abstract

Obesity is characterized by specific changes in the composition of the gut microbiota (GM). Exercise can contribute to the modulation of GM. This is the first case study to analyze the composition and metabolism of the GM of an obese runner in a single‐stage mountain ultramarathon (MUM) with a mileage of 217 km. Fecal samples were collected 7 days before the race (T0), 15 min after the end of the race (T1), and 7 days after the end of the race (T2). GM composition was analyzed by real‐time PCR and shotgun sequencing. We observed a decrease in Bacillota/Bacteroidota ratio and α‐diversity after the race. After the 217‐km MUM, we observed a decrease in symbiont microorganisms and a notable increase in harmful bacteria. In conclusion, we found that the 217‐km MUM may have contributed to the intestinal dysbiosis of the obese runner.

## 1 INTRODUCTION

Physical exercise is being investigated as a non‐pharmacologic intervention to mitigate the pro‐inflammatory pathways associated with obesity (Calcaterra et al., 2022). Obesity correlates with gut microbiota (GM) changes, including changes in α‐diversity and the ratio between Bacillota and Bacteroidota ratio. This shift can increase energy production and central appetite. GM plays a crucial role in the regulation of systemic inflammation, body weight control and the production of metabolites that have anti‐inflammatory effects and improve intestinal permeability (Xiong et al., 2022).

In the current scientific literature, there is a report that endurance events over shorter distances (marathon and Olympic triathlon) may contribute to increasing the α‐diversity of obese athletes (Barton et al., 2021). On the other hand, an ultra‐endurance triathlon can influence the metabolism without changing the composition of GM (Grosicki et al., 2023). However, there is no description of the acute effects of ultramarathon competitions on the GM of obese runners. So, are the effects of this type of exercise beneficial for GM in obese individuals? Or could it “worsen” gut health?

In this study, we investigated the changes that occurred in the GM of an obese runner at the Brazil 135 Ultramarathon‐2021 edition (BR135). Based on the above, the focus and originality of this study was to investigate the modulation of GM composition (down to the species level) as a result of the ultramarathon in an obese runner and the resilience (in the last 7 days) of the GM.

## 2 MATERIALS AND METHODS

### 2.1 Experimental design

The local research ethics committee (Comitê de Ética em Pesquisa da Unicamp) granted approval for the study (number 4.179.685; July 29, 2020). One week before the race (T0), the participant informed consent was signed, a stool sample was collected, anthropometric measurements were taken, the food frequency questionnaire (FFQ) was completed, and the plasma lactate concentration was measured with a hand‐portable lactate analyzer (Accutrend® Plus, ROCHE) by the researcher. Immediately after the race finished (T1), the plasma lactate was verified. Another stool sample was at T1 and 1 week after its completion (T2). Additionally, the volunteer completed the International Physical Activity Questionnaire‐short version (IPAQ) applied to this last day.

The study was conducted around the Brazil 135 Ultramarathon‐2021 edition. This 217‐km foot race is a single‐stage mountain ultramarathon (217‐MUM) performed on dirt roads in the Mantiqueira Mountains‐Brazil, with a total positive and negative elevation change of 12,200 m.

### 2.2 Fecal sample collection, transportation, and storage

One month before the 217‐MUM, sterile collection bottles, a pair of gloves, and instructions for proper collection and storage of biological material were mailed to the athlete. Instructions for collecting samples were then given to the athlete by message and in person. All samples were transported on dry ice and then stored in the laboratory at −20°C for further analysis, as specified in the guidelines.

### 2.3 Participant

The participant is a 59‐year‐old male, with 122.90 kg, height of 1.66 m, BMI 44.60 kg/m^2^, 40.27% body fat, and have no metabolic and inflammatory bowel diseases. In T0, his plasma lactate concentration was 1.9 mmol/L. He finished the 217‐MUM in 105:34:36 h, in 113th place, with 117.40 kg and BMI 40.27 kg/m^2^, with 5.1 mmol/L of plasma lactate concentration. Although the participant had been sedentary and increased his body weight during preceding years, he was accepted to participate in the race given his robust sport curriculum (more than 25 years of racing experience and has participated in 10 ultramarathon) and for being physically active before the competition. At T0, the runner's diet consisted with 5329.32 kcal of 77.15% of carbohydrates, 12.05% of protein, 10.80% of fat, and 31.86 g of fibers per day. The T0 fecal sample was considered the “control” for metagenomics analysis.

### 2.4 Metagenomic shotgun sequencing

DNA extraction from each sample was performed using the ZymoBIOMICS DNA Miniprep Kit (Cat. No. D4300, Zymo Research) in strict accordance with the instructions provided by the manufacturer. Library preparation was performed using the Illumina DNA Prep Kit (Cat. No. 20018704, Illumina Inc., USA). The concentration of the resulting DNA library was determined using Picogreen (Cat. No. P7589, Invitrogen, USA). Sequencing of the samples was performed on a NextSeq1000 instrument (Illumina Inc., USA) using Illumina's standard primers from the manufacturer's kit. Paired‐end runs with 150 base pair reads were performed using the NextSeq 1000/2000 P1 reagent kit (Cat. No. 20050264, Illumina, USA). The quality of the reads was assessed using FASTQC 0.11.8 (Bolger; Lohse; Usadel, 2014).

Taxonomic profiling of the samples was performed with MetaPhlAn 3 (Blanco‐Míguez et al., 2023). Heatmaps were created using GraphPad Prism 9 (GraphPad Software, MA, USA) and OriginPro (OriginLab, MA, USA). The Shannon index was calculated using the alpha_diversity.py script from the Qiime2 pipeline (http://qiime.org/scripts/alpha_diversity.html).

### 2.5 Real‐time PCR analysis

Real‐time PCR analysis was performed to confirm the metagenomic sequencing results. DNA extraction from the samples was performed using the QIAamp PowerFecal Pro DNA Kit (#51804, QIAGEN Group, Hilden, DE) according to the protocol indicated by the manufacturer. DNA was quantified and checked for quality using a Nanodrop. Real‐time PCR was then performed according to the method described by Possemiers et al. (2006), with the selection of specific primers based on the study of Rinttila et al. (2004). The primer used for *Lactobacillus* spp. was developed before the reclassification of this genus.

## 3 RESULTS

**FIGURE 1:**
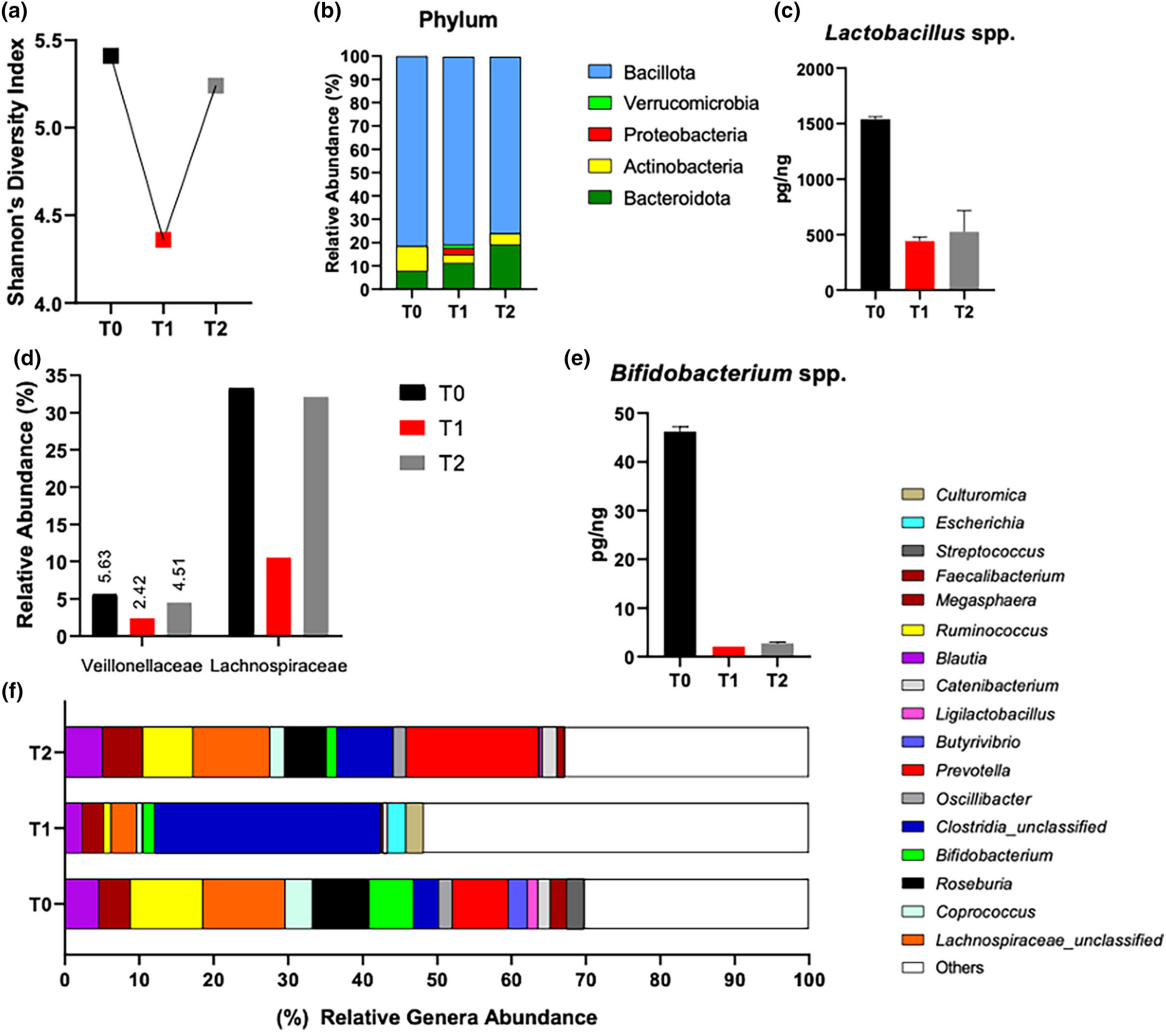
Different bacterial taxonomic levels of runner's gut microbiota (GM) obtained with metagenomic shotgun sequencing and Real‐time PCR analysis. (a) α‐diversity of bacteria species; (b) relative abundance of the phylum level; (c) quantity of *Lactobacillus* spp. DNA; (d) relative abundance of the Veillonellaceae and Lachonospiraceae families; (e) quantity of *Bifidobacterium* spp. DNA; (f) relative abundance of the genera level in gut microbiota changes. T0, seven days before the race; T1, immediately after the race; T2, seven days after the race. Data are expressed in relative abundance (%).

Immediately after the 217‐MUM the α‐diversity of the GM (Shannon diversity index) decreased by −19.32% compared to T0, while at T2, it returned close to baseline (Figure 1a). The ratio between Bacillota/Bacteroidota decreased over the course of the collections (T0: 1.05; T1: 0.71; T2: 0.39, respectively).

By metagenomic shotgun sequencing analysis, the phyla Pseudomonadota was only observed in T1, with relative abundances of 2.86% (Figure 1b). The bacterial family found in greater abundance in T0 was the Lachnospiraceae (33.13%) and, after the 217‐MUM, its relative abundance decreased by −68.28%, but in T2, it almost reached the initial value (32.13%). The same was observed for the Veillonellaceae family, which decreased in the bacterial percentage immediately after the race (−56.99%), as shown in Figure 1d.

As for the genus level in T1, we observed a strong increase in the relative abundance of *Clostridia* unclassified (+791.06%) and a decrease in *Prevotella* (−99.47%), *Roseburia* (−99%), *Ruminococcus* (−90.04%), *Ligilactobacillus* (previously classified as *Lactobacillus*) (−81.05%), *Coprococcus* (−80.46%), and *Bifidobacterium* (−73.25%) compared to T0, as shown in Figure 1f. However, at T2, *Clostridia* unclassified, Lachnospiraceae unclassified, *Faecalibacterium*, and *Blautia* returned near baseline levels.

Some bacterial genera were analyzed by real‐time PCR, which confirmed the results obtained by shotgun sequencing. The genera *Lactobacillus* (Figure 1c) and *Bifidobacterium* (Figure 1e) showed a sudden decrease immediately after the 217‐MUM and remained stable in T2.

**FIGURE 2:**
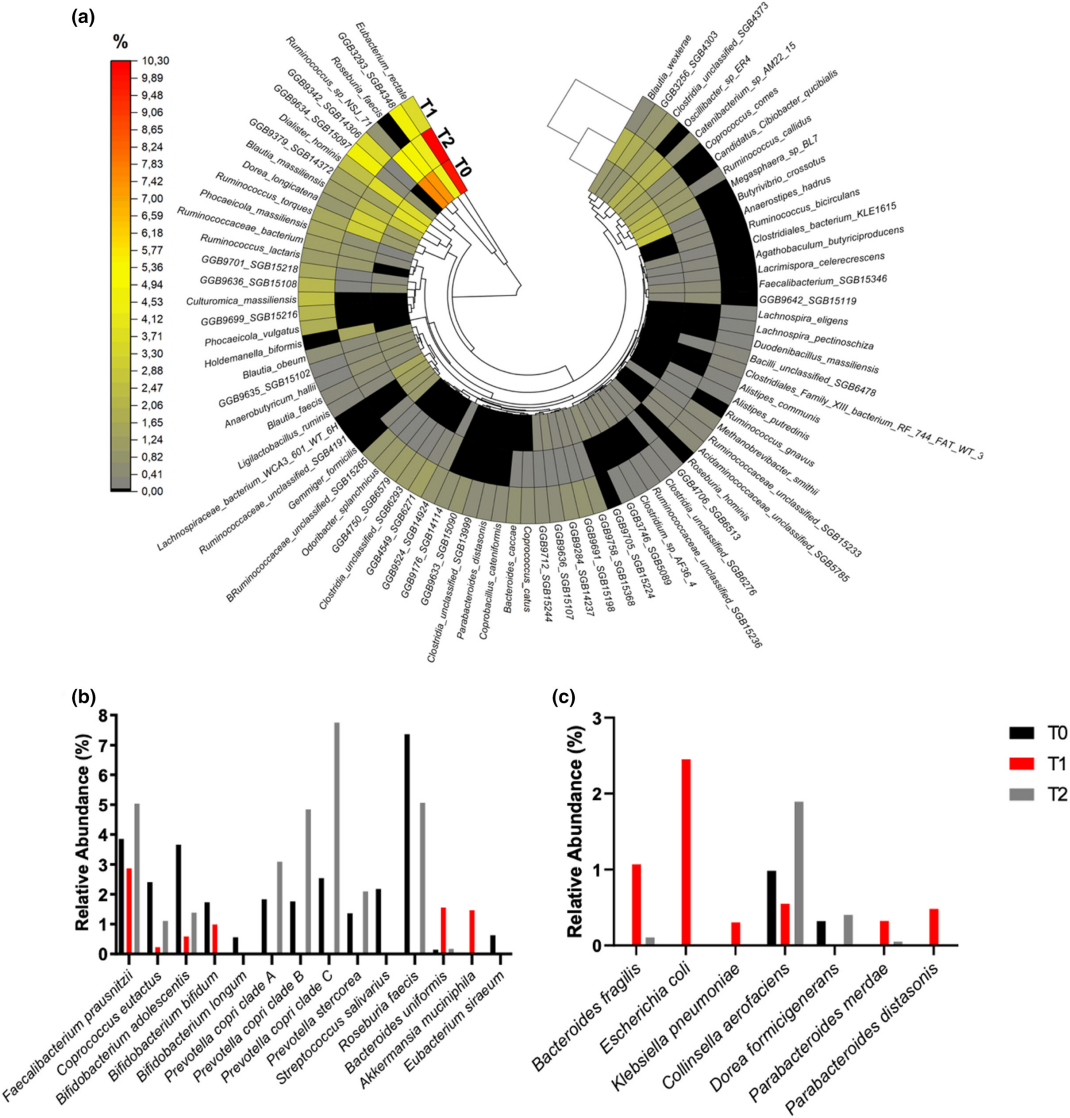
Relative abundance of specifics bacterial taxa in runner's gut microbiota. (a) Polar heatmap of the bacterial species found in the intestinal microbiota of runner obtained by metagenomic shotgun sequencing analysis; (b) “friendly” species in the gut microbiota (GM) of the obese runner; (c) “unfriendly” species in the GM of the obese runner. T1, immediately after the race; T2, seven days after the race. The data are expressed in relative abundance (%). Source adapted from Wang et al. (2021) and Wegierska et al. (2022).

At T0, the most abundant species in the GM of the athlete in question were *Eubacterium rectale* (9.87%), *Roseburia faecis* (7.36%), and *Ruminococcus* sp. *NSJ 71* (7.61%). However, at T1, they all showed a decrease (−64.45%, −100, and − 90.78%, respectively) (Figure 2a). At the same time, we also observed a considerable increase in the proportion of *Ruminococcus lactaris* and *Bacteroides uniformis* (+2963.70% and + 1009.57%, respectively). In addition, we also detected the reduced health‐related bacterial taxa after the 217‐MUM (Figure 2b). Furthermore, other bacterial species potentially harmful to human health were found (e.g., *Escherichia coli*, *Bacteroides fragilis*, and *Klebsiella pneumoniae*), as shown in Figure 2c. However, at T2, some harmful and human health‐related bacteria returned to near baseline levels (e.g., *Faecalibacterium prausnitzii, R. faecis, E. rectale, Prevotella copri* clades A, B, and C). In contrast, *Akkermansia muciniphila*, *Eubacterium siraeum*, *Bifidobacterium bifidum*, and *Bifidobacterium longum* were no longer detected.

## 4 DISCUSSION

The acute effect of the 217‐MUM promoted expressive changes in the composition and metabolism of the obese runner's GM; there was a decrease in “friendly” taxa; an increase in “unfriendly” bacteria. These factors can promote an inflammatory profile and have detrimental effects on the runner's health. These previously undocumented changes highlight the lack of in‐depth studies in this area.

Metagenomic sequencing revealed a reduction in α‐diversity in the obese runner during the race, which contrasts with previous research on faster ultramarathon runners (Grosicki et al., 2019; Saragiotto et al., 2023). Barton et al. (2021) showed an increase in α‐diversity in an obese runner after a 42‐km run and in another obese participant after an Olympic triathlon. In this sense, Estaki et al. (2016) have shown that greater GM diversity correlates positively with better cardiorespiratory fitness in humans. Our results could be related to the distance, the type of sport and the runner's diet at the race, as they differ from the others endurance modalities. According to Hills et al. (2019), the reduction in α‐diversity can be linked to diseases. Bacillota/Bacteroidota ratio decreased in the course of our study. However, Sato and Suzuki (2022), who studied ultramarathon runners, and Ley et al. (2006), who studied obese individuals, found higher proportions of this ratio in their work. It is suggested that bacteria from the phylum Bacillota can more efficiently extract energy from food, suggesting possible effects on obesity and athletic performance (Turnbaugh et al., 2006). In this sense, Grosicki et al. (2019) showed a significant increase in this ratio after a 163‐km ultramarathon in a world‐class ultramarathon runner (BMI = 22.1 kg/m^2^ and body fat = 17.2%).

At T1, the Pseudomonadota phyla became evident (2.86%). Grosicki et al. (2019) identified Pseudomonadota (~16%) after WSER. Healthy individuals normally present a lower proportion of Pseudomonadota compared to obese individuals (Shin et al., 2015). This phylum is a biomarker for dysbiotic gut and includes several pathogens that are known to affect human health and can synthesize harmful metabolites (Coutinho‐Wolino et al., 2021).

We also observed a decrease (−56.99%) in Veillonellaceae and increased in plasma lactate concentration (2.7‐fold), which contrasts previous studies with marathon runners and fast ultramarathon runners (Grosicki et al., 2019; Saragiotto et al., 2023; Scheiman et al., 2019). It is possible that the speed of the obese runner could explain the reduction of these bacteria in T1.

Although the runner was on a high‐fiber diet (31.80 g/day) days before the race, it was observed a sharp decrease in “friendly” and SCFA‐producing bacteria, such as *Lactobacillus, Ligilactobacillus, Bifidobacterium, Prevotella, Roseburia*, and *Faecalibacterium*, indicating possible loss of homeostasis. This condition can increase intestinal permeability and contribute to the development of inflammatory bowel disease, cardiovascular disease, cancer, and other health conditions (Hills et al., 2019). Some species of these genera, referred to as probiotic and next generation probiotic strains, can be administered to maintain health (Chang et al., 2019; Indira et al., 2019).

We observed a decline of *F*. *prausnitzii*, one of the most abundant species in the GM of healthy individuals (~5% of total gut bacteria) (Leylabadlo et al., 2020). There was a great decrease in *Prevotella* and *Roseburia* (−99.47% and − 99%, respectively), which are considered biomarkers for health (Bresser et al., 2022; Maldonado‐Contreras et al., 2020; Tamanai‐Shacoori et al., 2017). The increase of *A. muciniphila* after the 217‐MUM, which is known to have mucolytic properties, can increase intestinal and systemic inflammation in a dysbiotic context by reducing the effectiveness of the intestinal barrier (Ottman et al., 2017).

*Eubacterium rectale* was the most prevalent bacterium in the GM of the obese runner. According to Wang et al. (2021), a high proportion of *E. rectale* is associated with the development of colon cancer. *Collinsella aerofaciens* and *Dorea formicigenerans* were present in high concentrations in the GM of the obese runner; these are considered biomarkers for obesity and may contribute to increased intestinal permeability (Companys et al., 2021; Raman et al., 2013).

After the 217‐MUM, the proliferation of “unfriendly” taxa such as *B*. *fragilis*, *E*. *coli*, *K*. *pneumoniae, Parabacteroides merdae*, and *Parabacteroides distasonis* was observed in the GM of the obese runner, which was not identified in T0. *B. fragilis* produce an enzyme with proteolytic activity that can affect the intestinal barrier, leading to morphological and functional changes (Nakano & Avila‐Campos, 2004). Metabolites synthesized by this species pose a pathogenic risk (Wang et al., 2021). *E. coli* was found abundantly (~2.5% relative abundance) in the runner's GM at the post‐race time. Studies associate high levels of this taxon with obesity, inflammatory bowel disease, and type 2 diabetes (Gao et al., 2015; Karlsson et al., 2012). *K. pneumoniae*, which is highly virulent, causes upper respiratory tract infections and is resistant to antibiotics. It is also responsible for 11.8% of hospital‐acquired pneumonia and can cause inflammation in the colon by secreting toxins (Ashurst & Dawson, 2018; Kaur et al., 2018). *P. merdae* and *P. distasonis* were identified in higher proportions post‐race. High levels of these bacteria are associated with inflammatory bowel disease and cancer (Cui et al., 2022; Ezeji et al., 2021; Wegierska et al., 2022).

The present study presents a number of limitations, such as the inability to collect biological material at the beginning and during the race or to acquire data on nutrition and sleep patterns during the race. These data are of great importance as they can influence the composition and metabolism of this athlete's GM.

The results observed in the obese runner markedly differ from those documented in previous studies of faster ultramarathon runners with a eutrophic BMI. The ultramarathon, along with factors such as the runner's BMI, race distance, diet, sleep patterns, and environmental conditions, may have contributed to a state of imbalance of the runner's GM. The observed outcomes are specific to the single case and cannot be broadly applied; however, these data may contribute to characterize the acute effects of ultra‐endurance exercise on the GM ecosystem in obese individuals.

## AUTHOR CONTRIBUTIONS

Conception and design of research: Giulio Kai Saragiotto, Adriane Elisabete Costa Antunes, and Taisa Belli. Performed experiments: Giulio Kai Saragiotto, Milena Merizzi de Oliveira, Luiz Felipe Valter de Oliveira, Fernando Moreira Simabuco and Taisa Belli. Analyzed data: Giulio Kai Saragiotto, Luiz Felipe Valter de Oliveira, Fernando Moreira Simabuco, Fernanda Campos Freire, Nayara Kastem Scharlack, and Adilson Sartoratto. Interpreted results of the experiments: Giulio Kai Saragiotto, Fernando Moreira Simabuco, Fernanda Campos Freire, and Adilson Sartoratto. Drafted, edited and revised manuscript: Giulio Kai Saragiotto, Katia Sivieri, Adriane Elisabete Costa Antunes, and Taisa Belli. All authors approved the final version of manuscript.

## FUNDING INFORMATION

This work was supported by the CNPq (Grants: 303772/2022–0), CAPES code 001 and FAEPEX/UNICAMP (2160/23).

## CONFLICT OF INTEREST STATEMENT

The authors declared that no conflicts of interest.

## ETHICS STATEMENT

The study was approved by the Comitê de Ética em Pesquisa da Unicamp (n. 4.179.685; July 29, 2020).

## Data Availability

https://www.ncbi.nlm.nih.gov/bioproject/PRJNA1045616/.
